# Not All Brain Bleeds Are Equal: An Etiology-First Framework for Stroke Prevention After Intracerebral Hemorrhage

**DOI:** 10.7759/cureus.116830

**Published:** 2026-09-24

**Authors:** FNU Abdul Rehman, Muhammad Zeeshan, Muhammad Usman Siddique, Imran Brohi

**Affiliations:** 1 Internal Medicine, Mayo Hospital, Lahore, PAK; 2 Internal Medicine, Continental Medical College, Lahore, PAK

**Keywords:** atrial fibrillation, cerebral amyloid angiopathy, direct oral anticoagulants, intracerebral hemorrhage, left atrial appendage closure, magnetic resonance imaging, stroke prevention

## Abstract

Stroke prevention after intracerebral hemorrhage (ICH) in patients with atrial fibrillation (AF) requires balancing cardioembolic protection against recurrent cerebral bleeding. Randomized evidence indicates that direct oral anticoagulants (DOACs) substantially reduce ischemic stroke compared with no anticoagulation but may increase recurrent ICH in susceptible patients. Importantly, previous ICH does not represent a uniform biological risk. Deep hemorrhage related to hypertensive arteriopathy, lobar hemorrhage associated with cerebral amyloid angiopathy (CAA), mixed small-vessel disease, and hemorrhage from a treatable cause have different recurrence profiles. Magnetic resonance imaging (MRI) markers, including cerebral microbleed distribution, cortical superficial siderosis, convexity subarachnoid hemorrhage, lacunes, and enlarged perivascular spaces, can help characterize cerebral bleeding vulnerability, although no single marker should determine treatment. This editorial proposes an etiology-first, MRI-informed framework for selecting among restarting a DOAC, considering left atrial appendage closure (LAAC), and withholding long-term antithrombotic therapy. LAAC is not a risk-free alternative because it carries procedural risks and generally requires short-term antithrombotic treatment. Until prospective trials validate phenotype-specific pathways, ICH etiology and MRI phenotype should inform multidisciplinary, individualized, and preference-sensitive decisions.

## Editorial

Introduction

Consider two survivors of intracerebral hemorrhage (ICH) with atrial fibrillation (AF) and similar CHA₂DS₂-VASc scores. The first is 68 years old and experienced a single deep thalamic hemorrhage during uncontrolled hypertension, with only a few deep cerebral microbleeds. The second is 80 years old and has a spontaneous lobar ICH, multiple strictly lobar microbleeds, and disseminated cortical superficial siderosis. Although both have "previous ICH," their underlying cerebral vasculopathies and risks of recurrence are unlikely to be equivalent.

AF continues to confer substantial cardioembolic risk after the acute hemorrhage stabilizes. Withholding anticoagulation therefore does not eliminate harm but exchanges treatment-related bleeding for potentially preventable ischemic events. PRESTIGE-AF illustrated this tension: direct oral anticoagulants (DOACs) substantially reduced ischemic stroke but increased recurrent ICH compared with no anticoagulation [1]. These opposing effects argue against a uniform strategy.

Conventional cardiovascular scores offer limited guidance. CHA₂DS₂-VASc estimates thromboembolic risk, whereas commonly used bleeding scores do not adequately characterize brain-specific vulnerability associated with hemorrhage location, cerebral amyloid angiopathy (CAA), cerebral microbleeds, or cortical superficial siderosis [2]. Stroke prevention after ICH should therefore begin by establishing why and where the brain bled. We propose an etiology-first framework, informed by magnetic resonance imaging (MRI), to guide selection among restarting a DOAC, left atrial appendage closure (LAAC), and withholding long-term antithrombotic therapy.

Randomized evidence has clarified the trade-off, not solved it

Randomized evidence has replaced observational reassurance with a clearer, but less comfortable, account of competing risks. PRESTIGE-AF randomized 319 survivors of spontaneous ICH and AF to a DOAC or no anticoagulation. Over a median follow-up of 1.4 years, DOAC therapy reduced first ischemic stroke (hazard ratio (HR) 0.05, 95% confidence interval (CI) 0.01-0.36), with rates of all ischemic stroke events of 0.83 versus 8.60 per 100 patient-years. However, for first recurrent ICH, DOAC therapy did not meet the prespecified noninferiority criterion (HR 10.89, 90% CI 1.95-60.72). The rates of all ICH events were 5.00 versus 0.82 per 100 patient-years in the DOAC and no-anticoagulation groups, respectively [1]. PRESTIGE-AF therefore demonstrated substantial ischemic efficacy without establishing universal net benefit.

COCROACH was a prospective individual-participant-data meta-analysis of randomized trials that included 412 patients enrolled in SoSTART, APACHE-AF, NASPAF-ICH, and the relevant subgroup of ELDERCARE-AF. Oral anticoagulation reduced ischemic major adverse cardiovascular events (HR 0.27, 95% CI 0.13-0.56), while its effect on any stroke or cardiovascular death remained uncertain (HR 0.68, 95% CI 0.42-1.10). The effect on hemorrhagic major adverse cardiovascular events was also statistically uncertain (HR 1.80, 95% CI 0.77-4.21), because the confidence interval included both no effect and potentially important harm [3]. SoSTART and APACHE-AF were individually underpowered for definitive safety conclusions but showed substantial vascular-event rates under either strategy.

The evidence supports neither therapeutic nihilism nor routine anticoagulation. DOACs prevent ischemic stroke, but average treatment effects cannot identify the patient in whom cerebral bleeding vulnerability outweighs that benefit. That judgment requires understanding why the brain bled.

First ask why the brain bled

The anatomical label "ICH" encompasses biologically distinct disorders. Deep hemorrhages involving the basal ganglia, thalamus, pons, or cerebellum commonly reflect hypertensive arteriopathy. Long-standing hypertension, deep cerebral microbleeds, lacunes, and perforator-territory small-vessel disease strengthen this attribution. Because blood pressure is a major modifiable determinant of recurrence, a patient with a single deep hemorrhage, sustained blood-pressure control, and few additional hemorrhagic markers may represent a comparatively more defensible phenotype for reconsidering a DOAC [2]. However, this interpretation remains inferential: randomized evidence has not validated this specific phenotype as a treatment-selection criterion or demonstrated that it identifies patients with a favorable net benefit from DOAC therapy. It should therefore inform individualized multidisciplinary assessment rather than be regarded as a declaration of safety.

Lobar ICH in an older adult raises concern for CAA. Strictly lobar microbleeds, cortical superficial siderosis, convexity subarachnoid hemorrhage, and severe perivascular spaces in the centrum semiovale support this diagnosis under the Boston criteria version 2.0 [4]. CAA is a diffuse, persistent vasculopathy rather than a transient precipitant that resolves with the index hematoma. Disseminated cortical superficial siderosis or recurrent lobar hemorrhage may identify particularly high bleeding vulnerability, which anticoagulation could further amplify.

Some patients have both lobar and deep hemorrhagic markers, suggesting mixed small-vessel disease, overlapping pathologies, or diagnostic uncertainty. They should not be assigned mechanically to either pathway. Secondary causes must also be distinguished from primary small-vessel disease. Vascular malformations, tumors, cerebral venous thrombosis, vasculitis, and reversible coagulation abnormalities have different recurrence trajectories. A definitively treated structural lesion may leave less residual vulnerability than diffuse, progressive small-vessel disease. Etiological investigation therefore helps define whether stroke prevention is reasonable, hazardous, or potentially futile.

MRI as a treatment-stratification tool

MRI can transform "previous ICH" into a more informative bleeding phenotype. Hemorrhage location and microbleed distribution provide the initial distinction: predominantly deep findings favor hypertensive arteriopathy, whereas strictly lobar findings support CAA. Susceptibility-sensitive sequences can reveal cortical superficial siderosis, convexity subarachnoid hemorrhage, and previous silent hemorrhage, all of which may indicate greater vulnerability [2,4].

Microbleed count alone should not become an arbitrary treatment threshold. A numerical cutoff ignores distribution, accompanying abnormalities, and the underlying vasculopathy. Several deep microbleeds in severe hypertension may not carry the same biological implications as fewer strictly lobar microbleeds accompanied by disseminated cortical superficial siderosis. Pattern and context may therefore be more informative than burden alone [1,3]. White-matter hyperintensities, lacunes, and enlarged perivascular spaces further characterize small-vessel disease but rarely determine treatment independently. Deep lacunes and basal ganglia perivascular spaces favor hypertensive arteriopathy, whereas severe centrum semiovale perivascular spaces may support CAA. Mixed-location microbleeds increase diagnostic uncertainty.

MRI should inform treatment rather than function as an autonomous decision rule. Randomized trials have not established reliable treatment effects within individual imaging phenotypes. Findings must be integrated with ICH etiology, cardioembolic risk, blood-pressure control, renal function, frailty, previous ischemic events, and patient preferences. MRI does not provide binary permission for anticoagulation; it reveals the cerebral substrate on which treatment will act [2,4].

Matching phenotype to treatment

An etiology-first framework should guide clinical reasoning rather than prescribe treatment. The strongest case for restarting a DOAC may be a patient with a single deep hypertensive ICH, controlled blood pressure, high cardioembolic risk, and few or no lobar hemorrhagic markers. Even in this comparatively favorable phenotype, treatment should follow confirmation of hematoma stability, sustained blood-pressure control, dose adjustment for renal function, and withdrawal of unnecessary antiplatelet therapy [1,2].

Conversely, recurrent lobar ICH, probable CAA, or disseminated cortical superficial siderosis should prompt strong caution regarding long-term anticoagulation [2,4]. LAAC may be considered when cardioembolic risk remains substantial, but neither LAAC nor withholding anticoagulation should follow automatically from a diagnosis of CAA. When cerebral bleeding vulnerability is extreme or expected benefit is limited by severe dependency, short life expectancy, or modest embolic risk, choosing neither a DOAC nor LAAC may be reasonable. Such a decision does not imply therapeutic inaction; rigorous blood-pressure control and management of other vascular risk factors remain essential.

A treated structural lesion or reversible cause represents a separate pathway. After definitive treatment and imaging confirmation of low residual bleeding risk, reconsidering a DOAC may be appropriate. Mixed or uncertain phenotypes warrant multidisciplinary review involving stroke neurology, cardiology, neuroradiology, and the patient. Expert MRI interpretation may be more informative than another conventional risk score [1].

LAAC should not be portrayed as a bleeding-free substitute for anticoagulation. It carries procedural complications, device-related thrombus, peri-device leak, and a requirement for short-term antithrombotic therapy, for which the optimal post-ICH regimen remains uncertain. CLOSURE-AF randomized 912 patients with AF and high risks of stroke and bleeding to LAAC or physician-directed medical therapy. The primary composite of stroke, systemic embolism, cardiovascular or unexplained death, and major bleeding occurred in 16.83% and 13.27%, respectively (adjusted HR 1.28, 95% CI 1.01-1.62); noninferiority was not demonstrated [5]. However, CLOSURE-AF was not restricted to patients with previous ICH, and its findings cannot be directly extrapolated to determine the comparative safety or effectiveness of LAAC in the post-ICH population. The trial nevertheless provides a broader caution against assuming that LAAC is inherently safer than contemporary medical therapy. Dedicated randomized evidence in ICH survivors is required. Until then, etiological assessment and individualized multidisciplinary decision-making should precede either withholding anticoagulation or pursuing LAAC.

What the evidence still cannot tell us

Future trials should distinguish deep from lobar ICH, apply validated CAA criteria, and prespecify microbleeds and cortical superficial siderosis. Comparisons among DOACs, LAAC, and no long-term antithrombotic therapy should use disability-weighted outcomes because ischemic stroke and recurrent ICH are not clinically equivalent. The safest antithrombotic regimen after LAAC in ICH survivors also requires dedicated study.

Previous ICH should trigger phenotyping, not therapeutic paralysis. Deep hypertensive hemorrhage, probable CAA, mixed small-vessel disease, and treated structural bleeding are not interchangeable. Until prospective trials validate a formal algorithm, etiology and MRI phenotype should inform, but not dictate, the choice among a DOAC, LAAC, and neither.
